# Supplementary figures and images for: Morphometric responses of two zooxanthellate octocorals along a water quality gradient in the Cuban northwestern coast
Source: PLoS One. 2023 Aug 18;18(8):e0290293. doi: 10.1371/journal.pone.0290293 (PMC10437867; doi:10.1371/journal.pone.0290293)

A

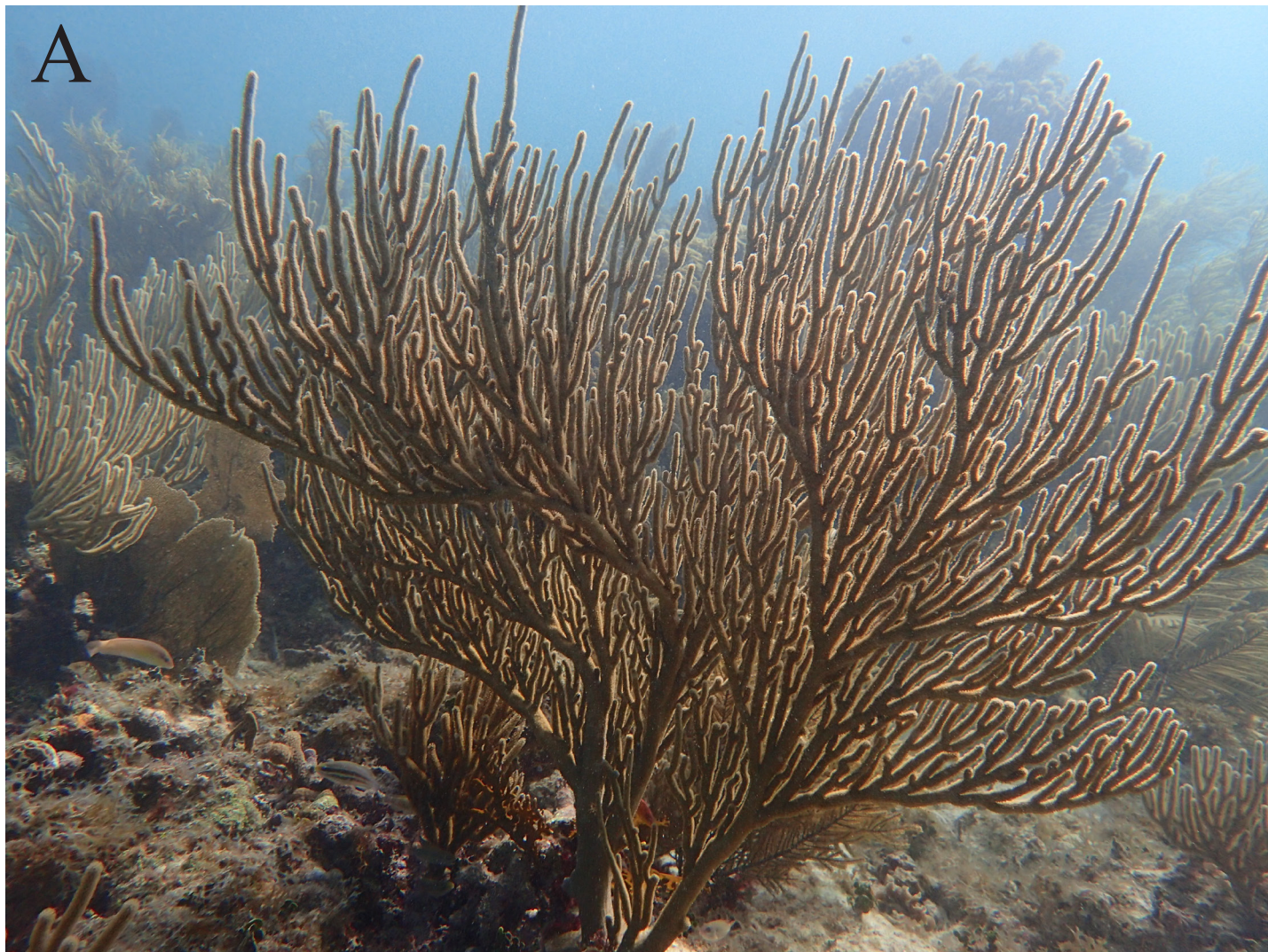

B

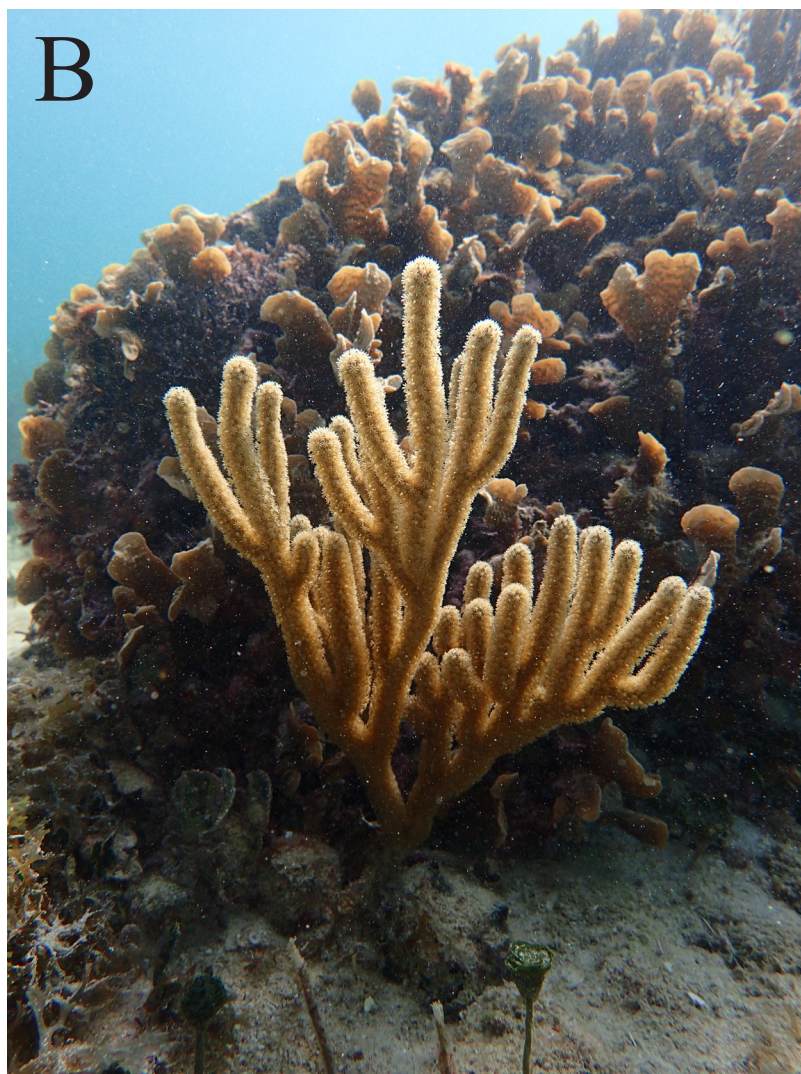

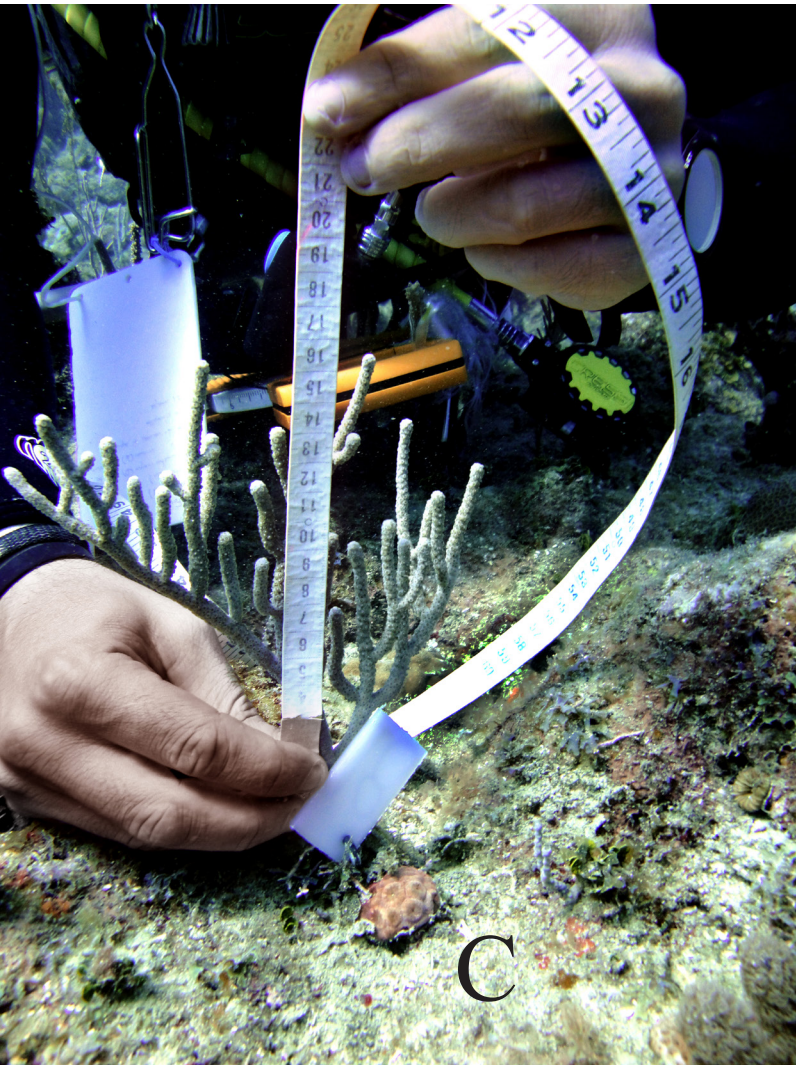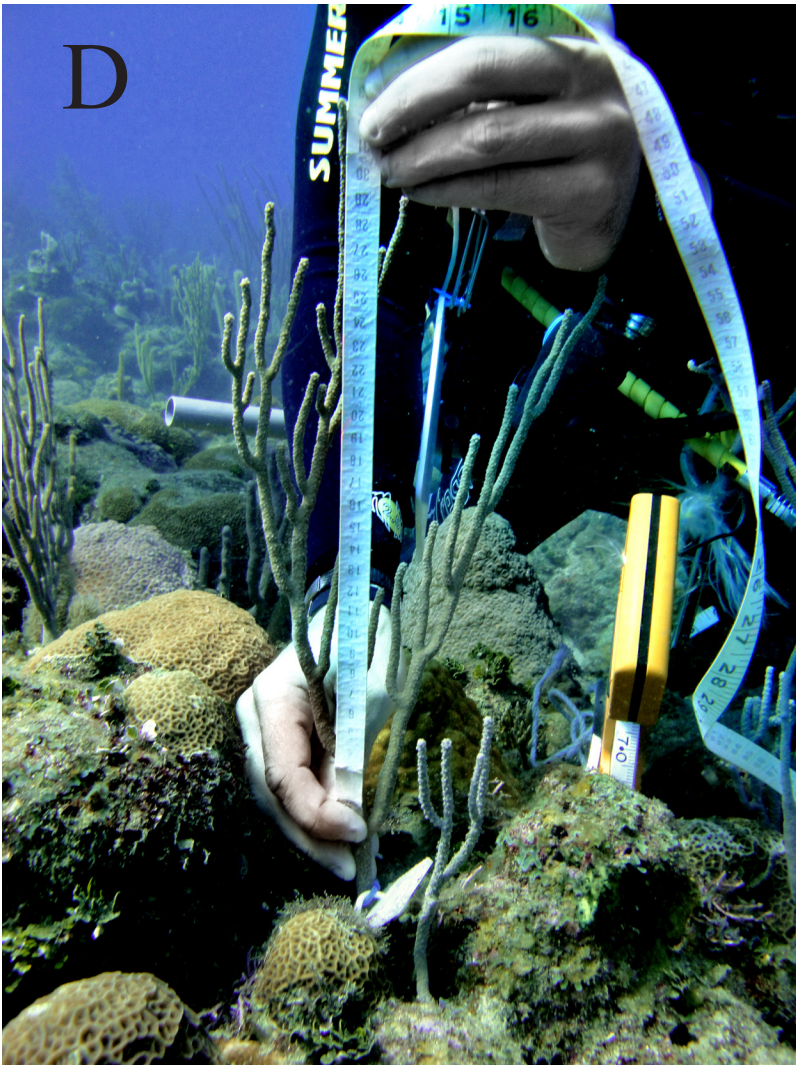

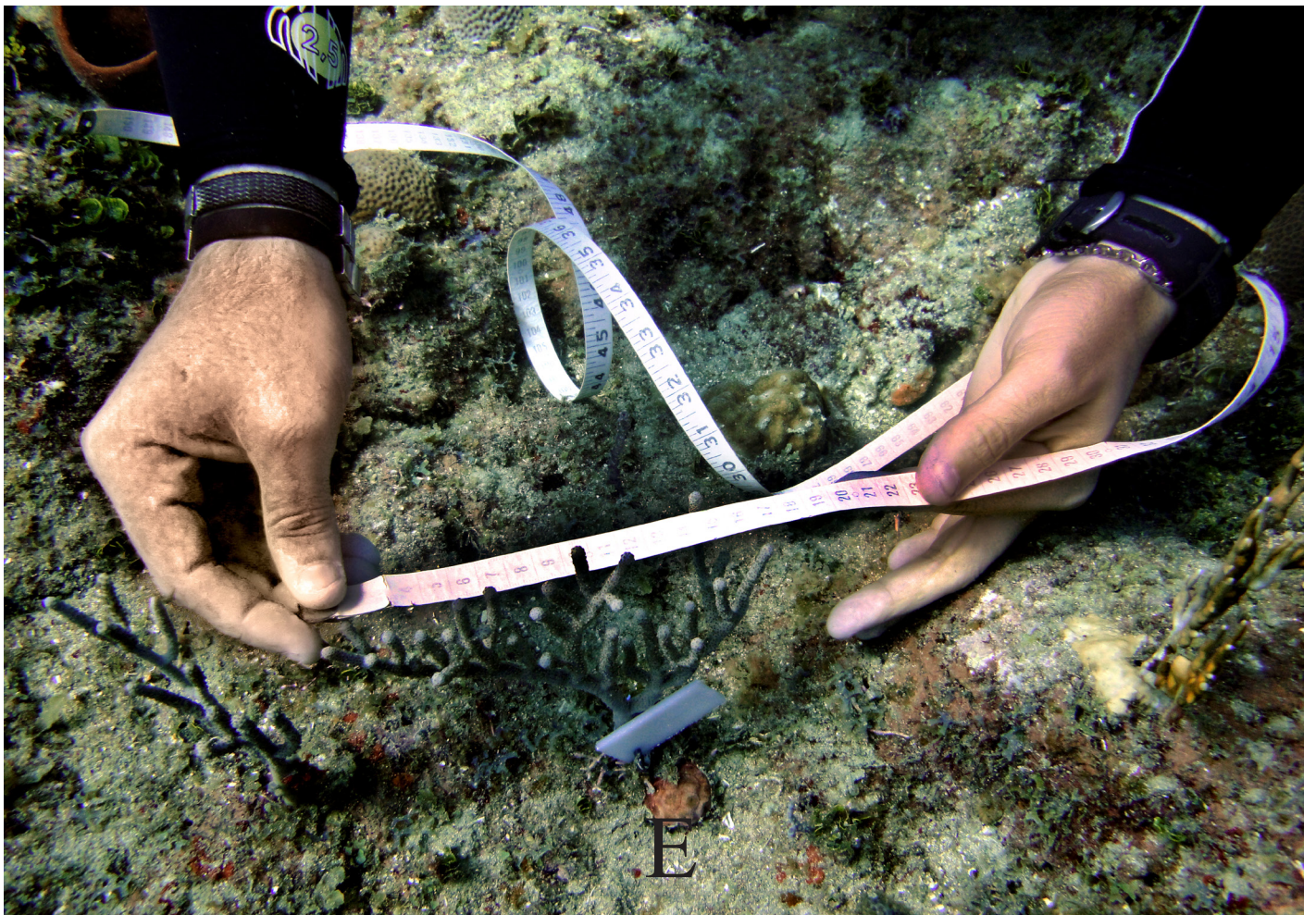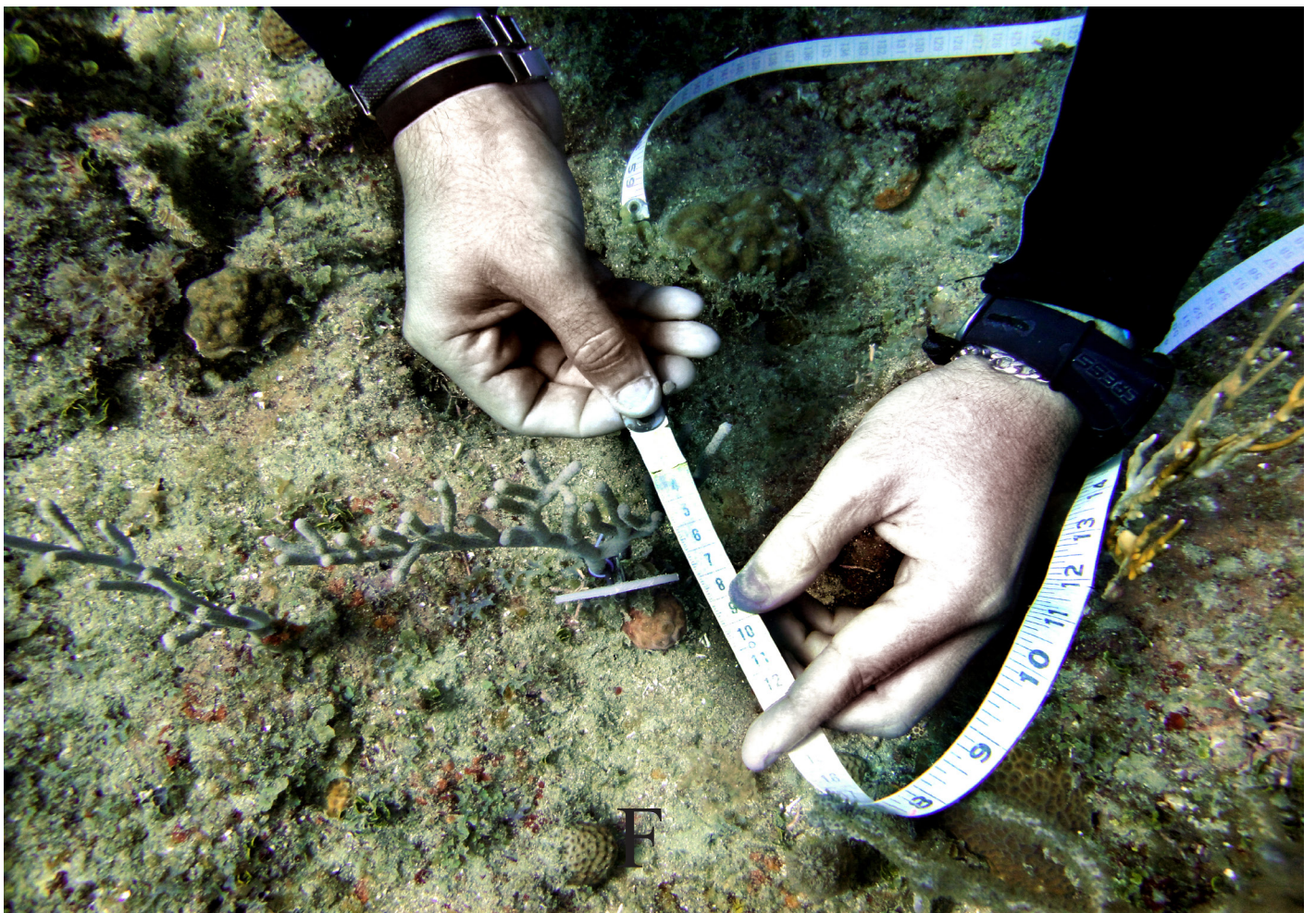

Supplement: S1 Fig — Photos of Eunicea flexuosa (A), Plexaura kükenthali (B), height from the holdfast to the colony apex (C), height from first branching to the colony apex (D), maximum diameter (E), and minimum diameter (F) of the colony from a top view. Photos credits: José Espinosa (C, D, E and F) and Néstor Rey-Villiers (A and B). (PDF) [file pone.0290293.s001.pdf]

Standard error accumulated

*Eunicea flexuosa*

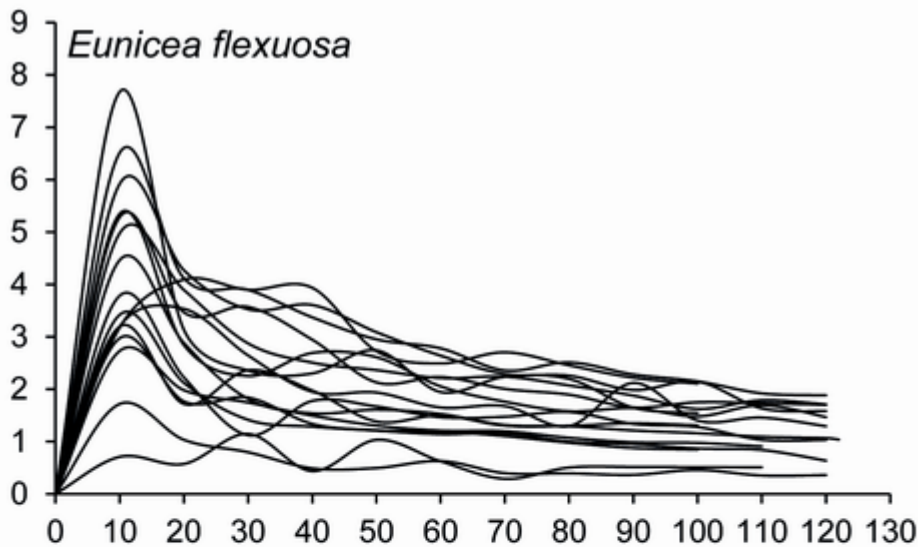

*Plexaura kükenthali*

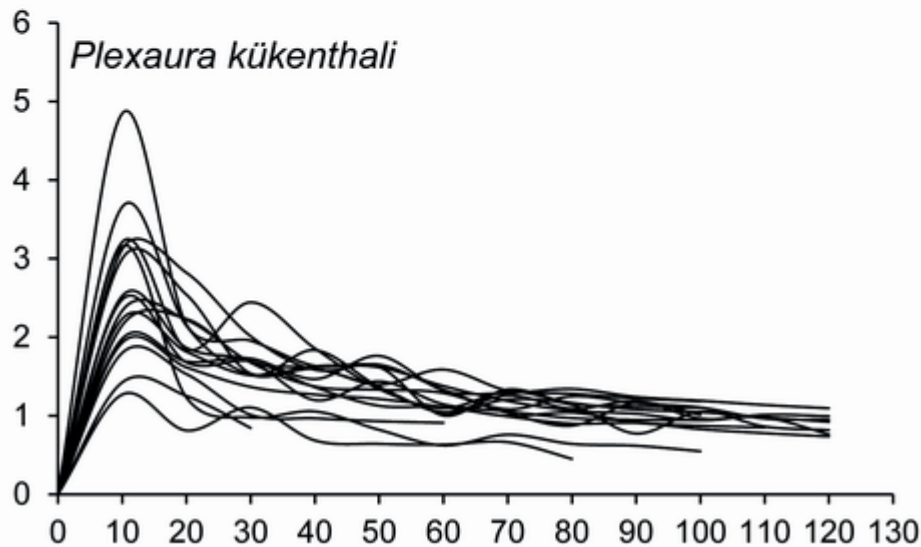

Number of colonies

Supplement: S2 Fig — (PDF) [file pone.0290293.s002.pdf]

Relative frequency (%)

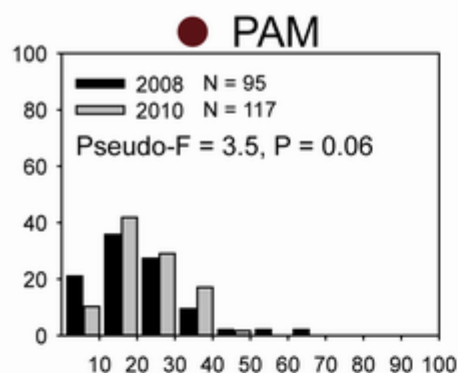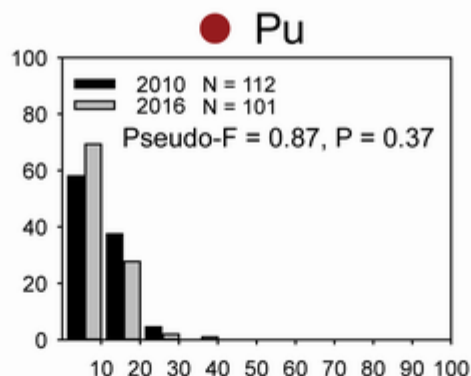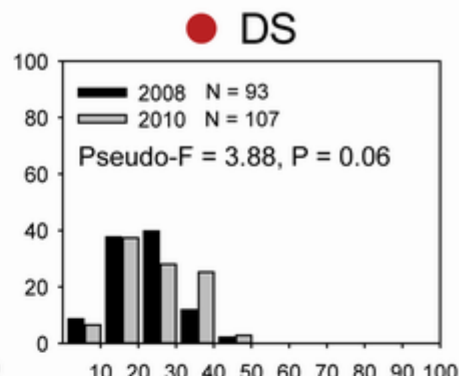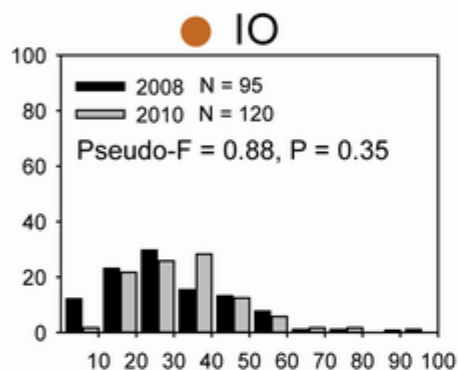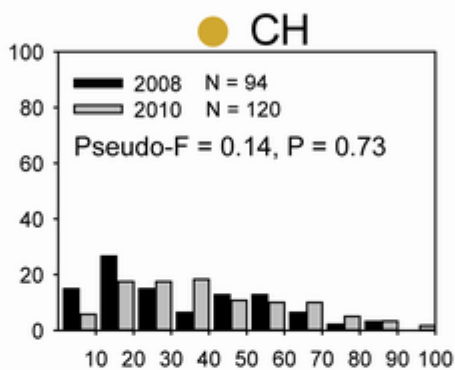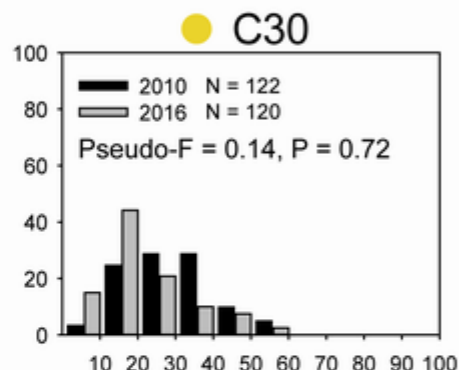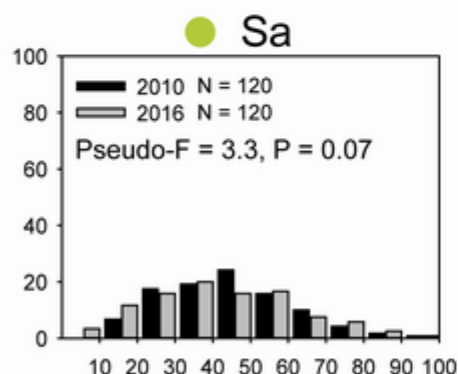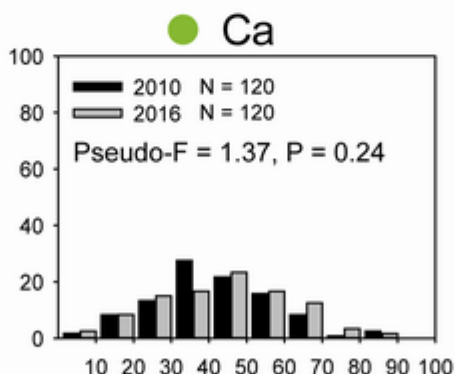

Size intervals (cm)

● PAM ● Pu ● DS ● IO ● CH ● C30 ● Sa ● Ca

Better water quality

Supplement: S3 Fig — The statistics results of PERMDISP test are shown. N: sampling sizes. Codes of the sampling sites are presented in Fig 1. (PDF) [file pone.0290293.s003.pdf]

Relative frequency (%)

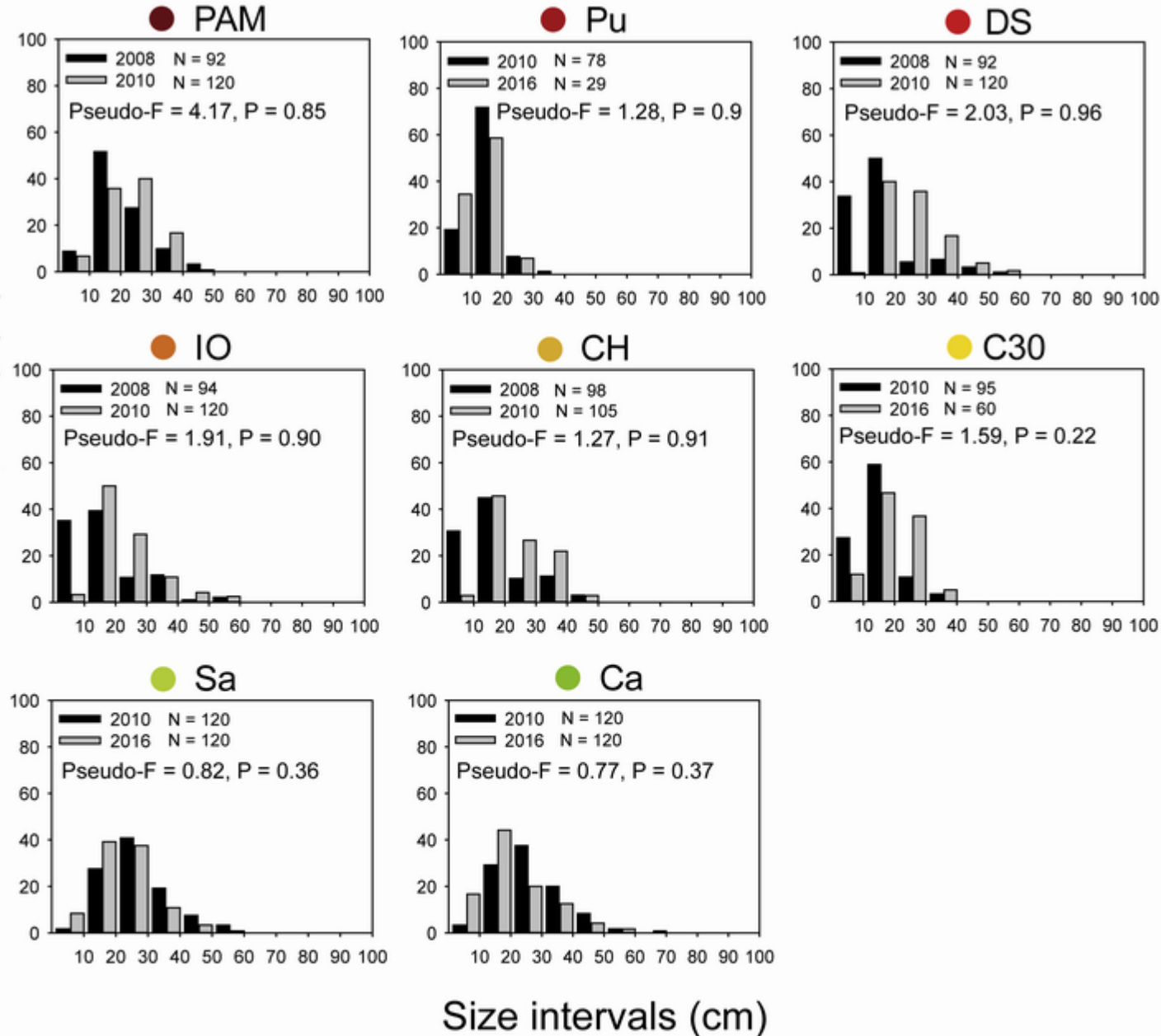

● PAM ● Pu ● DS ● IO ● CH ● C30 ● Sa ● Ca

Better water quality

Supplement: S4 Fig — The statistics results of PERMDISP test are shown. N: sampling sizes. Codes of the sampling sites are presented in Fig 1. (PDF) [file pone.0290293.s004.pdf]
