# Supplementary material for: Morphometric responses of two zooxanthellate octocorals along a water quality gradient in the Cuban northwestern coast
Source: PLoS One. 2023 Aug 18;18(8):e0290293. doi: 10.1371/journal.pone.0290293 (PMC10437867; doi:10.1371/journal.pone.0290293)
Supplement: S2 Table — FC: fecal coliform bacteria, HB: heterotrophic bacteria, TC: total coliform bacteria, SR: sulfate-reducing bacteria, FE: fecal streptococcal bacteria, DIN: dissolved inorganic nitrogen, and HSI: hydrodynamic stress index. The correlations in black indicate P values < 0.05. (PDF) [file pone.0290293.s006.pdf]

S2 Table. Pearson´s correlation between the morphometric indicators of *E. flexuosa* and the microbiological, hydrochemical, and physical variables and stable nitrogen isotopes of *E. flexuosa*. FC: fecal coliform bacteria, HB: heterotrophic bacteria, TC: total coliform bacteria, SR: sulfate-reducing bacteria, FE: fecal streptococcal bacteria, DIN: dissolved inorganic nitrogen, and HSI: hydrodynamic stress index. The correlations in black indicate P values < 0.05.

Variables	Morphometric indicators of *E. flexuosa*				
Microbiological	Height (cm)	Maximum diameter (cm)	Number of terminal branches/colony	Cover index	H/D ratio
FC (MPN/100 mL)	**-0.81**	**-0.84**	**-0.79**	**-0.68**	**0.7**
HB (CFU/mL)	-0.47	-0.53	**-0.71**	-0.64	0.45
TC (MPN/100 mL)	**-0.84**	**-0.85**	**-0.83**	**-0.73**	**0.71**
SR (MPN/100 mL)	-0.07	-0.22	-0.39	-0.3	0.23
FE (MPN/100 mL)	**-0.69**	**-0.70**	**-0.64**	**-0.59**	0.53
Hydrochemical					
NH_4^+ (µmol/L)	**-0.74**	**-0.82**	**-0.84**	**-0.67**	**0.94**
NO_3^- + NO_2^- (µmol/L)	**0.77**	**0.81**	**0.8**	0.54	**-0.85**
DIN (µmol/L)	-0.43	-0.52	-0.57	-0.57	**0.71**
PO_4^{3-} (µmol/L)	-0.05	-0.23	-0.22	-0.29	0.28
Salinity (PSU)	0.28	0.31	0.51	0.23	-0.52
Physical					
Horizontal visibility (m)	**0.88**	**0.91**	**0.86**	**0.78**	**-0.76**
Bottom-sediment accumulation	**-0.66**	**-0.67**	**-0.78**	**-0.8**	0.49
HSI (%)	-0.59	-0.46	-0.25	-0.3	0.27
Stable nitrogen isotopes ($\delta^{15}N$)					
$\delta^{15}N$ in tissue of *E. flexuosa* (‰)	**-0.74**	**-0.83**	**-0.88**	**-0.67**	**0.82**
