# Supplementary material for: Morphometric responses of two zooxanthellate octocorals along a water quality gradient in the Cuban northwestern coast
Source: PLoS One. 2023 Aug 18;18(8):e0290293. doi: 10.1371/journal.pone.0290293 (PMC10437867; doi:10.1371/journal.pone.0290293)
Supplement: S3 Table — FC: fecal coliform bacteria, HB: heterotrophic bacteria, TC: total coliform bacteria, SR: sulfate-reducing bacteria, FE: fecal streptococcal bacteria, DIN: dissolved inorganic nitrogen, and HSI: hydrodynamic stress index. The correlations in black indicate P values < 0.05. (PDF) [file pone.0290293.s007.pdf]

S3 Table. Pearson´s correlation between the morphometric indicators of *P. kükenthali* and the microbiological, hydrochemical, and physical variables and stable nitrogen isotopes of *P. kükenthali*. FC: fecal coliform bacteria, HB: heterotrophic bacteria, TC: total coliform bacteria, SR: sulfate-reducing bacteria, FE: fecal streptococcal bacteria, DIN: dissolved inorganic nitrogen, and HSI: hydrodynamic stress index. The correlations in black indicate P values < 0.05.

Variables	Morphometric indicators of *P. kükenthali*				
Microbiological	Height (cm)	Maximum diameter (cm)	Number of terminal branches/colony	Cover index	H/D ratio
FC (MPN/100 mL)	-0.42	-0.42	**-0.67**	-0.37	0.66
HB (CFU/mL)	-0.18	-0.20	-0.29	-0.16	0.38
TC (MPN/100 mL)	-0.38	-0.4	**-0.63**	-0.36	**0.63**
SR (MPN/100 mL)	0.40	0.43	0.13	0.45	-0.19
FE (MPN/100 mL)	-0.33	-0.38	-0.63	-0.33	0.66
Hydrochemical					
NH_4^+ (µmol/L)	-0.53	-0.37	-0.41	-0.39	0.26
$NO_3^- + NO_2^-$ (µmol/L)	0.54	0.43	0.51	0.41	-0.40
DIN (µmol/L)	-0.28	-0.14	-0.11	-0.20	-0.04
PO_4^{3-} (µmol/L)	0.14	0.09	-0.17	0.04	-0.24
Salinity (PSU)	0.46	0.17	-0.09	0.08	-0.22
Physical					
Horizontal visibility (m)	0.47	0.52	**0.77**	0.49	**-0.67**
Bottom-sediment accumulation	-0.28	-0.34	-0.47	-0.28	**0.69**
HSI (%)	**-0.74**	**-0.85**	**-0.79**	**-0.83**	**0.82**
Stable nitrogen isotopes ($\delta^{15}N$)					
$\delta^{15}N$ in tissue of *P. kükenthali* (‰)	-0.34	-0.24	-0.4	-0.17	0.43
