# Supplementary material for: Morphometric responses of two zooxanthellate octocorals along a water quality gradient in the Cuban northwestern coast
Source: PLoS One. 2023 Aug 18;18(8):e0290293. doi: 10.1371/journal.pone.0290293 (PMC10437867; doi:10.1371/journal.pone.0290293)
Supplement: S4 Table — (PDF) [file pone.0290293.s008.pdf]

S4 Table. Mean growth rate (cm/year) in height (95% CI) of *E. flexuosa* and *P. kükenthali* at sites influenced by discharges from polluted river basins and sites not impacted by those basins.

River basins	Sites	Impacted sites	*E. flexuosa*	*P. kükenthali*	References
Havana Bay	PAM	Yes	3.6 (2.9-4.3)	4.1 (3.1-5.0)	Rey-Villiers and Sánchez [1]
Quibú River	DS	Yes	2.7 (2.4-3.0)	1.2 (0.9-1.5)	Unpublished data of N. Rey-Villiers
Salado River	Sa	No	1.7 (1.4-2.1)	1.8 (1.3-2.4)	Rey-Villiers and Sánchez [1]
There are no nearby basins within 4 km	Ca	No	1.8 (1.5-2.2)	1.1 (0.7-1.5)	Rey-Villiers and Sánchez [1]

Supporting information references

1. Rey-Villiers N, Sánchez A. ¿Puede la contaminación orgánica afectar la tasa de crecimiento de los octocorales en el Caribe? Gayana. 2018; 82 (2): 166-170. http://dx.doi.org/10.4067/S0717-65382018000200166
